# Regulation of Nucleotide Excision Repair by Nuclear Lamin B1

**DOI:** 10.1371/journal.pone.0069169

**Published:** 2013-07-24

**Authors:** Veronika Butin-Israeli, Stephen A. Adam, Robert D. Goldman

**Affiliations:** The Department of Cell and Molecular Biology, Northwestern University Feinberg School of Medicine, Chicago, Illinois, United States of America; University of Virginia, United States of America

## Abstract

The nuclear lamins play important roles in the structural organization and function of the metazoan cell nucleus. Recent studies on B-type lamins identified a requirement for lamin B1 (LB1) in the regulation of cell proliferation in normal diploid cells. In order to further investigate the function of LB1 in proliferation, we disrupted its normal expression in U-2 OS human osteosarcoma and other tumor cell lines. Silencing LB1 expression induced G1 cell cycle arrest without significant apoptosis. The arrested cells are unable to mount a timely and effective response to DNA damage induced by UV irradiation. Several proteins involved in the detection and repair of UV damage by the nucleotide excision repair (NER) pathway are down-regulated in LB1 silenced cells including DDB1, CSB and PCNA. We propose that LB1 regulates the DNA damage response to UV irradiation by modulating the expression of specific genes and activating persistent DNA damage signaling. Our findings are relevant to understanding the relationship between the loss of LB1 expression, DNA damage signaling, and replicative senescence.

## Introduction

The nuclear lamins are type V intermediate filament proteins found primarily within the nucleus of metazoan cells. The lamins play important roles in providing mechanical support and shape to the nucleus and in regulating many nuclear functions including DNA replication, Pol II transcription, DNA repair, mitotic spindle formation, response to oxidative stress, and chromosome positioning [1]. However, the mechanisms by which lamins mediate these functions remain largely unknown. There are two types of lamins expressed in cells of vertebrates, the A-types, comprised of lamins A and C (LA and LC), and the B-types, lamin B1 (LB1) and lamin B2 (LB2). LA and LC are expressed in developmentally regulated patterns from a single gene by alternative splicing. In contrast LB1 and LB2 are expressed from two different genes, with at least one B-type lamin being expressed in all cell types throughout development and differentiation [2].

Hundreds of mutations have been identified in *LMNA*, the gene encoding the A-type lamins, causing a spectrum of rare diseases known as laminopathies [3]. There is evidence that mutated forms of A-type lamins exert their deleterious effects on cells by multiple mechanisms including altering the interaction of the lamins with lamin-binding proteins, causing telomere dysfunction, disrupting the epigenetic regulation and organization of chromatin, and altering gene expression [4]. Additional changes that are associated with mutations in A-type lamins include activation of DNA repair regulating factors and check point kinases, which possibly contribute to impaired cell cycle progression and replication arrest [5], [6]. Furthermore, in affected cells an accumulation of unrepaired DNA has been observed due to delayed recruitment of DNA repair proteins to the DNA damage sites [7]. In contrast to the numerous mutations in A-type lamins, mutations in the B-type lamins are rare. The only known disease involving LB1 is adult-onset autosomal dominant leukodystrophy (ADLD), a progressive demyelinating disease caused by the overexpression of LB1 in neurons due to either a gene duplication or a mutation in the *LMNB1* promoter [8]. Further analyses of ADLD patients' cells has revealed that this overexpression causes the disorganization of inner nuclear membrane proteins and chromatin, and the down regulation of myelin gene expression [9]. Studies of mouse models made null for LB1 or expressing a truncated form of LB1 show defects in organogenesis, in particular, the brain [10]–[12]. However, skin keratinocytes, hepatocytes, or embryonic stem cells (ESC) derived from these mice proliferate normally, have no obvious nuclear abnormalities, and show only minor changes in their transcription profile in comparison to wild-type cells [12], [13]. The expression of the B-type lamins has not been extensively explored in cancer cells, although decreases in LB1 expression have been reported in neoplasms of the gastrointestinal tract [14] and in some subtypes of lung cancer [15]. In light of these findings and the paucity of LB1 mutations, it appears that the levels of LB1 in the nucleus need to be tightly controlled.

Recently, we and others have shown that LB1 expression is reduced during normal replicative senescence in cultured human diploid fibroblasts and in aged mouse and human tissue [16]–[18]. However, conflicting findings from several groups on the effects of experimentally induced LB1 depletion or overexpression on cell proliferation and senescence in cultured normal fibroblasts suggests that the mechanisms by which LB1 regulates cell proliferation are complex [17], [19]. In order to further investigate the role of LB1 in regulating proliferation, we altered its expression in tumor cell lines by shRNA mediated silencing to determine the requirement for LB1 expression in cells with abnormal cell cycle controls. Our findings demonstrate that silencing LB1 expression in tumor cells rapidly induces cell cycle arrest and causes a delayed response to UV-induced DNA damage repair.

## Materials and Methods

### Cell culture and silencing

The human U-2 OS cell line (ATCC, HTB-96) was cultured in McCoy's 5a medium supplemented with 10% fetal bovine serum (FBS) and 100 units/mL penicillin and 100 ug/mL streptomycin. The MCF7 cell line (ATCC, HTB-22) was cultured in modified Eagle's medium (MEM) supplemented with 10 ug/mL insulin, 0.1 mM non-essential amino acids and 1 mM sodium pyruvate. HCC 1937, MDA-MB-231, MDA-MB-435 and HeLa S3 cells were obtained from ATCC and cultured in RPMI-1640, Leibovitz's L-15 and Dulbecco's modified Eagle's medium (DMEM), respectively. All culture media were supplemented with 10% fetal bovine serum (FBS) and 100 units/mL penicillin and 100ug/mL streptomycin. All cells were maintained at 37°C in a humidified atmosphere and 5% CO2. For silencing LB1 expression, cells were transfected with the previously described silencing vector by electroporation (220 V 960 mF) [17], [20].

### Immunoblotting

Total cell lysates were prepared with Laemmli buffer [21]. The protein concentration of samples was determined using the BCA protein assay kit (Thermo Scientific). The protein samples were separated by SDS-PAGE on 10% gels and transferred to nitrocellulose. Primary antibodies used for immunoblotting were: mouse anti-LA/C (5G4), rabbit anti-LB1 [22], mouse anti-LB1/2 (2B2); rabbit anti-CHK1, anti-pCHK1 (S345), anti-CHK2, anti-pCHK2 (Cell Signaling); rabbit anti-ATM, rabbit anti-pATM (Epitomics), mouse anti-p53 (DO-1), rabbit anti-ATR, rabbit anti-pATR, mouse anti-PCNA (PC10), rabbit anti-DDB1, goat anti-CSB, rabbit anti-53BP1 (Santa Cruz Biotechnology); rabbit anti-pRPA32 (Bethyl Labs); mouse anti γH2AX (JBW301, Millipore); mouse anti-GAPDH (FF26A/F9, Biolegend, Inc.). Secondary antibodies conjugated with horseradish peroxidase (1 mg/mL; KPL) were used at a dilution of 1∶50,000 and the peroxidase activity was detected using the SuperSignal West Pico Chemiluminescence Detection kit (Thermo Scientific). Images were quantified with Kodak Molecular Imaging software.

### Immunofluorescence

U-2 OS cells grown on glass coverslips were fixed in methanol for 10 min at −20°C followed by permeabilization with 0.1% Triton X-100 in PBS for 10 min at 22°C. Primary antibodies used for immunofluorescence were mouse anti-LB1/2, rabbit anti-LB1 [22], rabbit anti-pRPA32 (Bethyl Labs), mouse anti- γH2AX (JBW301, Millipore), rabbit anti-DDB1 and rabbit anti-53BP1 (Santa Cruz Biotechnology). Secondary antibodies included goat anti mouse IgG-Alexa Fluor 488 and goat anti-mouse IgG-Alexa Fluor568 (Invitrogen). DNA was stained with 1 ng/mL Hoechst 33258 (Invitrogen). After staining, coverslips were mounted on slides in 20 mM Tris-Cl (pH 9.0) with 50% glycerol and 1% p-phenylenediamine (Sigma-Aldrich). Images were obtained with a Zeiss LSM 510 microscope using oil immersion objective lenses (PlanApochromat, 63X and 100X, 1.40 NA).

### BrdU labeling

Detection of DNA replication was carried out as described [22]. Cells were labeled with 10 mM BrdU (Sigma-Aldrich) in growth medium for 3 h at 37°C. BrdU-labeled DNA was detected with rabbit anti-BrdU (Sigma-Aldrich), followed by goat anti-rabbit IgG-Alexa Fluor 488 (Invitrogen).

### UV irradiation

Cultured cells were washed once with PBS and irradiated with 254 nm UVC using a Stratagene UV Stratalinker 1800 at a fluency of 20 J/m^2^ as detected by a calibrated UVC radiometer (UVC light meter 850010; Sper Scientific). Following irradiation, growth medium was replaced on the cells and they were stored in the incubator until needed.

### ELISA with a specific cyclobutane pyrimidine dimer antibody

We followed the procedure for the ELISA detection of cyclobutane pyrimidine dimers in genomic DNA as previously described [23]–[25]. Briefly, 1×10^6^ cells were cultured in 10 cm-dishes and irradiated with 20 J/m^2^ UVC (see above). Genomic DNA was purified from cells immediately after irradiation or 1, 2, 4, 8, 16, 24, 48 and 80 min later using a QIAamp Blood Kit (QIAGEN). CPD detection was performed on 20 ng of genomic DNA from each irradiated sample immobilized per well of a 96 well plate using the TDM-2 specific antibody (Cosmo Bio USA). The bound anti-CPD antibody was detected with peroxidase conjugated goat anti-mouse IgG (Zymed) and *o* -phenylenediamine (Sigma). The absorbance (OD) of reaction products was measured at 492 nm. The percent of CPDs remaining for each time point was calculated as the ratio of OD measured for specific time point and the OD from the DNA harvested immediately after irradiation. The experiment was repeated three times and the ELISA was performed in quadruplicate.

### Detection of DNA break

The detection of DNA breaks was performed with the in situ cell death detection kit and fluorescein (TUNEL; Roche) as previously described [26]. Negative-controls were prepared with only the labeling solution and positive-controls were assayed by adding DNase I (5 µg/mL) for 1 h at RT after Triton X-100 permeabilization. Cells were analyzed by FACS.

### Cell cycle analysis

For cell cycle analysis, LB1 silenced and control cells were collected by trypsinization at three and five days following transfection. For each analysis 1×10^6^ cells were washed once with PBS and fixed with 100% ethanol. The fixed cells were treated with RNaseA and 0.1% Triton-X100 in PBS for 3 h at RT, and stained with propidium iodide (PI). The cell cycle distribution of each sample was analyzed by fluorescence-activated cell sorting (FACS).

### Apoptosis Assay

To assay apoptosis we used the Annexin V Apoptosis Assay (Clontech, Mountain View, CA) following the manufacturer's instructions. Cells were counted by FACS following DNA staining with PI.

### Statistical analysis

We used the two-tailed Student's t-test for statistical analyses. All of the results presented are the mean ± standard deviation from three separate experiments. We considered results as significant when the p-value was equal to or less than 0.05.

### Gene expression analysis

Total cDNA was prepared from LB1 silenced and control cells as previously described [17]. The primers for gene specific analysis by qRT-PCR were obtained from Qiagen (QuantiTect Primer Assays kits). The qRT–PCR analyses were carried out with a LightCycler 480 Real-Time PCR instrument (Roche) using the LightCycler 480 SYBR Green Master Kit (Roche). Relative expression analysis was carried out using the LightCycler 480 Real-Time PCR software with GAPDH serving as the reference gene. Quantitative results are shown as the mean of 4 separate experiments. Using Student's t-test (p-value was equal to or less than 0.05), a change in the expression of a specific gene was considered significant if the “fold change” was greater than 1.7 or less than 0.6.

## Results

### LB1 silencing rapidly arrests the proliferation of tumor cells

**Figure 1:**
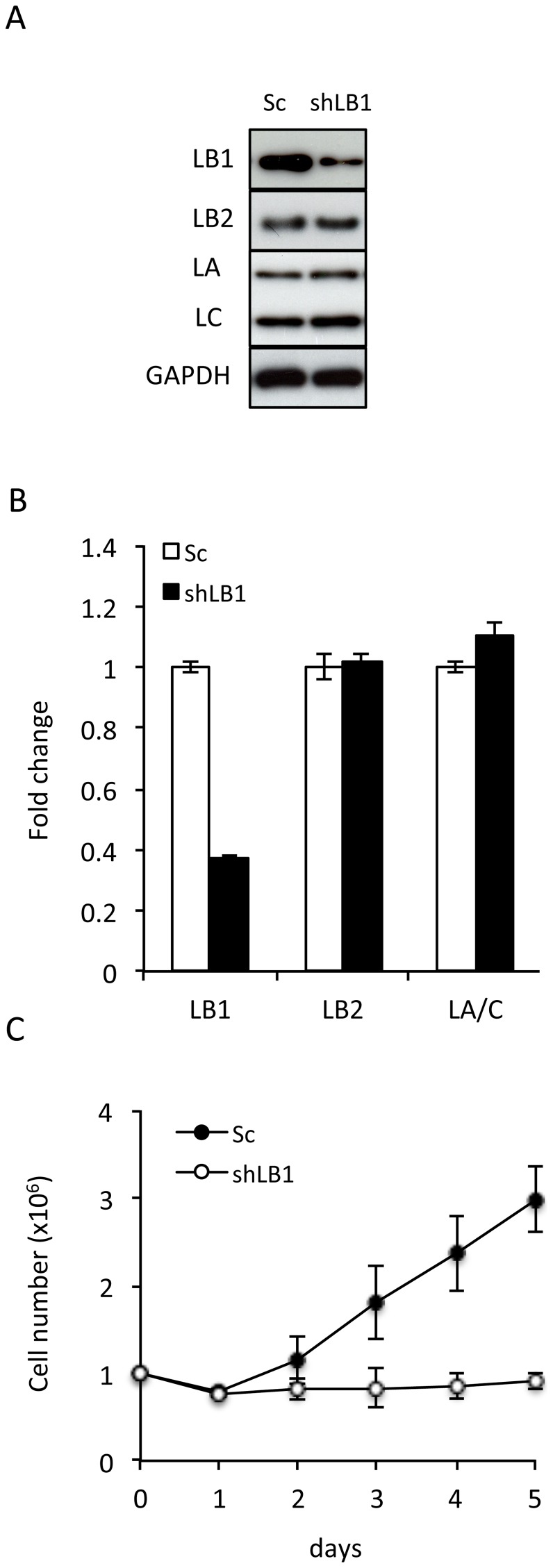
Transient silencing of LB1 induces growth arrest in U-2 OS cells. A. The protein levels of LB1, LB2, and LA and C were assayed by immunoblotting at day 3 after electroporation with the vector encoding shRNA (shLB1) or a scrambled sequence (Sc). B. Relative expression levels of *LMNB1*, *LMNB2*, and *LMNA* mRNA in cells were determined by qRT-PCR at day 3 after silencing using GAPDH as a reference gene. The error bars represent standard deviation of the mean (n = 5). C. Growth rate of shLB1 and Sc cells were compared for 5 days following silencing. Growth rate was evaluated as previously described [17] (n = 6, p = 5.24 ×10^−7^); error bars represent standard deviations.

Within three days following transient expression of a silencing vector targeting LB1 (shLB1) in the human osteosarcoma cell line U-2 OS, LB1 protein expression decreased by ∼75–80% as determined by immunoblotting; and its mRNA level was reduced by ∼65% as shown by qRT-PCR analyses (Fig. 1A, B). Silencing LB1had no significant effect on the expression levels of either LA/C or LB2 (Fig. 1A, B). A scrambled sequence shRNA (Sc) did not affect lamin expression and was used as a control throughout these studies (Fig. 1A, B). The decrease in LB1 levels after expressing the silencing vector was accompanied by a proliferation arrest (Fig. 1C). Similar decreases in proliferation rates were seen in other tumor cell lines following LB1silencing, including MDA-MB-35, MDA-MB-231, HCC 1937, HeLa and MCF 7 (Figure S1). The results obtained for all of the following experiments were similar for each of these cell lines; therefore we present only the data for U-2 OS cells.

### LB1 silencing causes cell cycle arrest in early G1

The cessation of proliferation in U-2 OS cells silenced for LB1 expression (Fig. 1C) was attributable to G1 cell cycle arrest as determined by FACS. The latter data showed that ∼87% of LB1 silenced cells were in G1 by day 3 following transfection with LB1 shRNA, compared to ∼55% of control cells [n = 4; p = 5.7×10^−3^]. Furthermore, FACS analysis also revealed that DNA replication, as assayed by BrdU incorporation, could be detected in only ∼5% of LB1 silenced cells in contrast to ∼28% of control cells [n = 3; p = 2.3×10^−3^].

**Figure 2:**
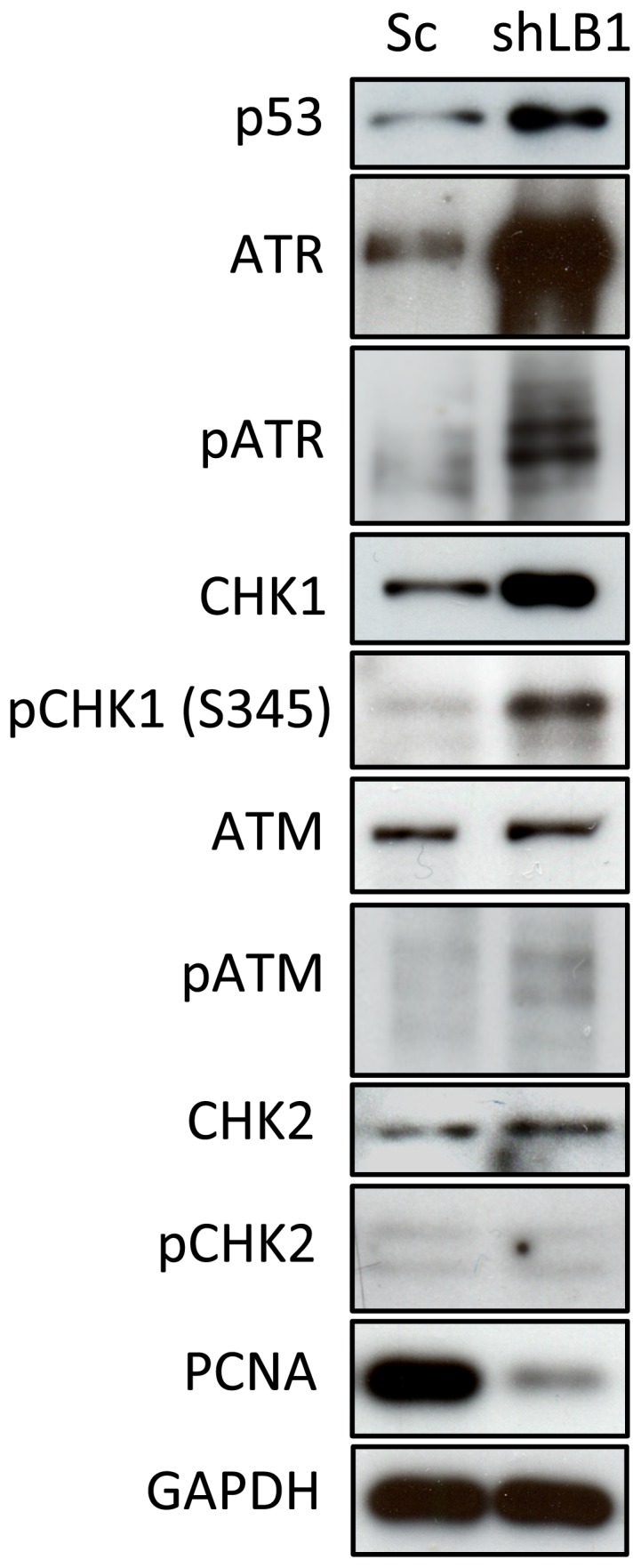
Activation of key signaling proteins that mediate early G1 arrest. Protein levels in silenced and control cells were detected by immunoblotting at day 3 after LB1 silencing. GAPDH served as a loading control. This experiment was repeated 4 times.

In order to analyze the G1 arrest in more detail, we carried out immunoblotting analyses of factors known to regulate progression through the G1 phase of the cell cycle including p53, ATM, ATR, CHK1 and CHK2 (Fig. 2). We detected a significant increase in p53 levels in LB1 silenced cells (Fig. 2). In addition, we found that the level of ATR increased and that both ATR and its substrate CHK1 showed increased phosphorylation demonstrating their activation [27], [28] (Fig. 2). Phosphorylation of ATM was not significantly altered and the phosphorylation of its downstream effector CHK2 could not be detected. Importantly, we also found that the expression of proliferating cell nuclear antigen (PCNA), a key component of the DNA replication machinery which is normally synthesized at the end of G1 [29], was reduced to ∼10% of controls (Fig. 2). Moreover, PCNA mRNA levels decreased to ∼30% of controls as determined by qRT-PCR. Taken together, these results show that LB1 silenced cells are arrested in the early G1 phase of the cell cycle.

### Silencing of LB1 causes increased sensitivity to UV irradiation

**Figure 3:**
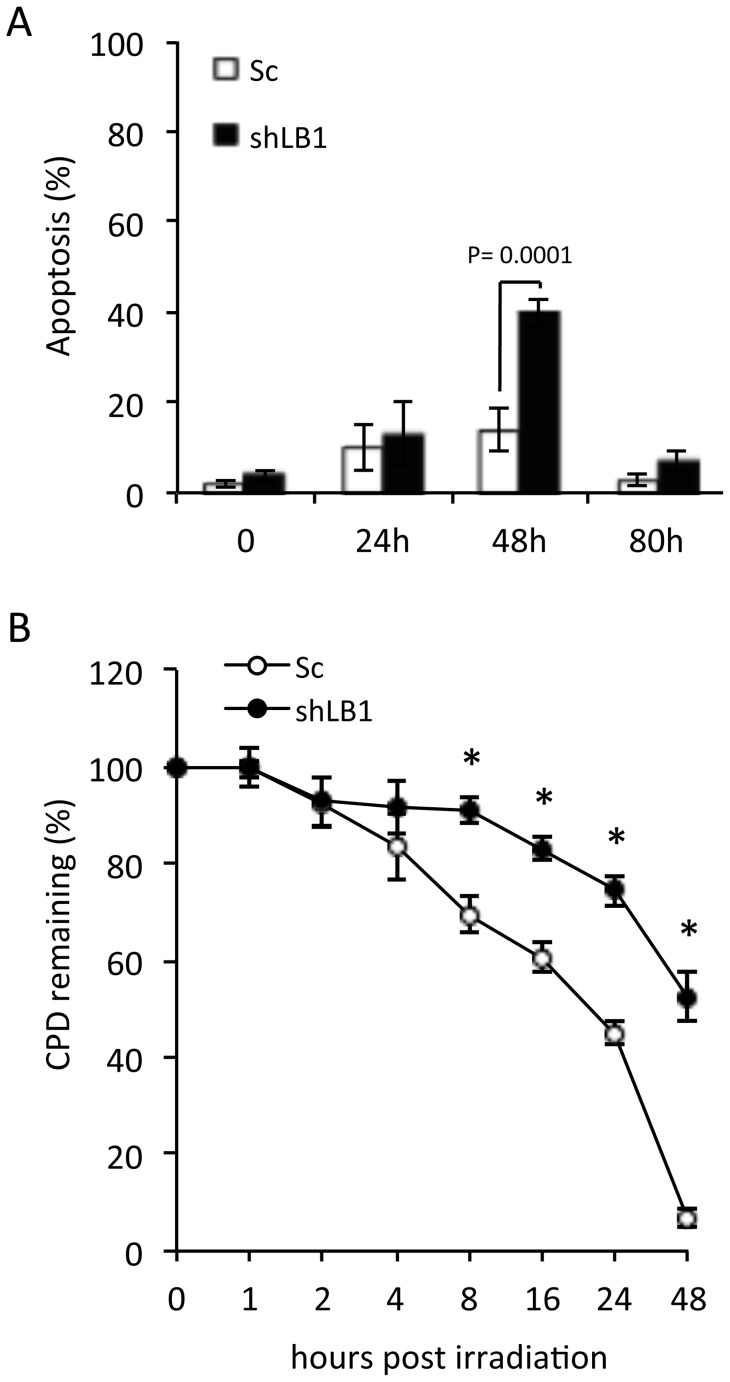
Delayed NER following UV irradiation of LB1 silenced cells. A. Detection of apoptosis in control (Sc) and LB1 silenced (shLB1) cells after 20 J/m^2^ UV irradiation. Irradiated cells were harvested at 0, 24, 48 and 80 hr after irradiation, stained for Annexin V/PI, and examined by FACS (n = 4); error bars represent standard deviations. B. Detection of CPD by ELISA. Silenced and control cells where irradiated with 20 J/m^2^ UV and harvested at the indicated times. CPD lesions were detected in genomic DNA by ELISA as described in Materials and Methods. The experiment was repeated 4 times, and each DNA sample was assayed by ELISA in quadruplicate; error bars represent standard deviations. Asterisks mark time points where significant differences in CPD clearance were observed between control (Sc) and LB1 silenced cells (shLB1): 8 hr p = 0.0057; 16 hr p = 0.001; 24 hr p = 0.0058; and 48 hr p = 0.0001.

The finding that the early G1 arrest induced by LB1 silencing was accompanied by the induction of p53 and activation of ATR (Fig. 2), suggested that DNA damage signaling or repair mechanisms might be defective [30]. However, we could not detect DNA damage within the nuclei of LB1 silenced cells using TUNEL [31], or by an increase in DNA damage foci as determined by indirect immunofluorescence staining with antibodies against phosphorylated replication protein A (pRPA32) [32], [33] and γH2AX [34] (Figure S2). The ability of the silenced cells to repair DNA damage was further assessed by irradiating cells with 20 J/m^2^ UV at day 3 after LB1 silencing and measuring the number of apoptotic cells at time intervals following irradiation. Control and LB1 silenced cells showed a similar rate of apoptosis at 24 hr after irradiation (Fig. 3A). However, at 48 hr, LB1 silenced cells had a much greater percentage of apoptotic cells (∼42%) when compared to control cells (∼18%). By 80 hr, only small numbers of apoptotic cells could be detected in both LB1 silenced (∼5%) and control (∼2%) cells. Importantly, 48 hr after irradiation control cells recovered and re-entered the cell cycle with ∼33% of cells in S phase, while the LB1 silenced cells that did not die by apoptosis remained arrested in G1 as determined by cell cycle analysis. The greater frequency of apoptosis after UV irradiation in LB1 silenced cells compared to controls, suggests that the UV induced DNA damage response and repair pathways are defective in LB1 silenced cells.

UV irradiation induces cyclobutane pyrimidine dimers (CPDs) that are removed by the nucleotide excision repair (NER) pathway [33]. In order to determine if LB1 silenced cells were deficient in NER, we used a quantitative ELISA to measure the CPD content of genomic DNA isolated from control and LB1 silenced cells following irradiation with 20 J/m^2^ UV [24], [25]. There was a significant delay of ∼7 hr before the initiation of CPD clearance in silenced cells as compared to control cells (Fig. 3B). Clearance of CPDs was essentially complete in control cells by 48 hrs post irradiation, but LB1 silenced cells required an additional 72 hr for complete CPD clearance. This delay in DNA repair is therefore the most likely cause of the significant increase in apoptosis in LB1 silenced cells at 48 hr following UV irradiation (Fig. 3A).

### Silencing of LB1 alters the expression of factors involved in DNA damage repair and signaling

The initial steps in the process of NER can be divided into two sub-pathways: global genomic NER (GG-NER) and transcription coupled NER (TC-NER). These pathways differ in the initial steps of DNA damage recognition: GG-NER is mediated by the damage-specific DNA binding proteins (DDB1/2) to recognize the lesions that occur throughout the genome, whereas TC-NER is initiated mainly by stalling of RNA Pol II at damage sites in actively transcribing genes, which recruits CSA (Cockayne syndrome A), and CSB (Cockayne syndrome B) [32], [33], [35], [36].

**Figure 4:**
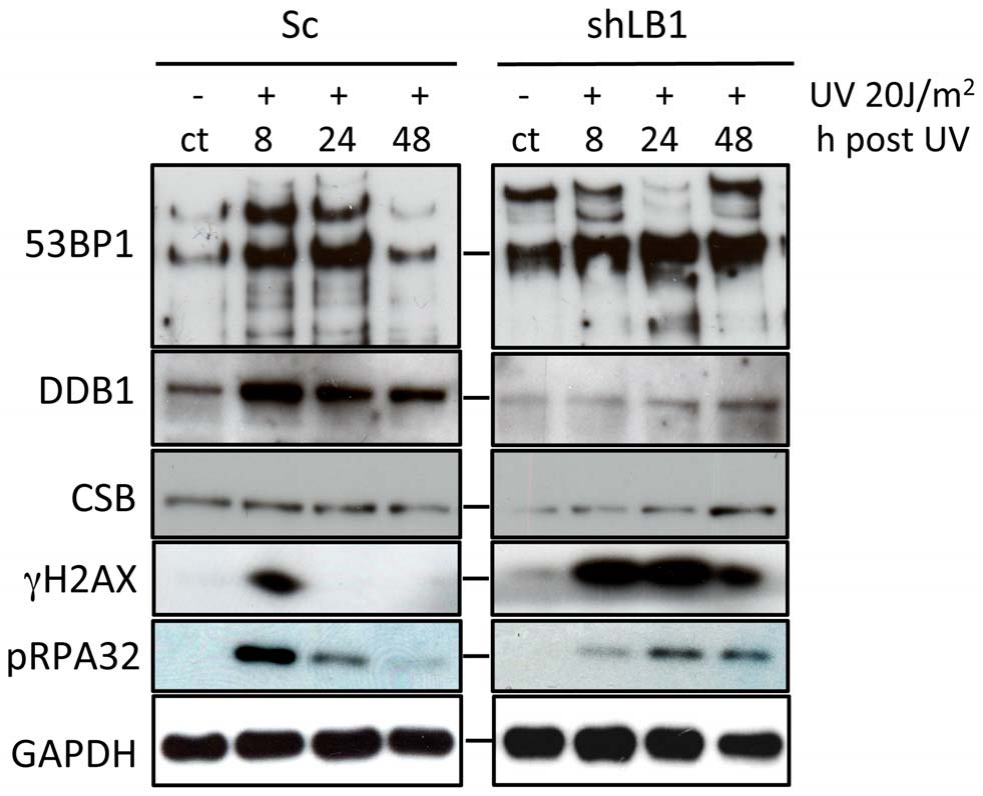
Immunoblotting of NER associated proteins. Sc and shLB1 cells were harvested 8, 24 and 48 hr after UV irradiation and total cell lysates were analyzed. Non-irradiated cells from the same transfections are labeled (ct). GAPDH detection served as loading control.

**Figure 5:**
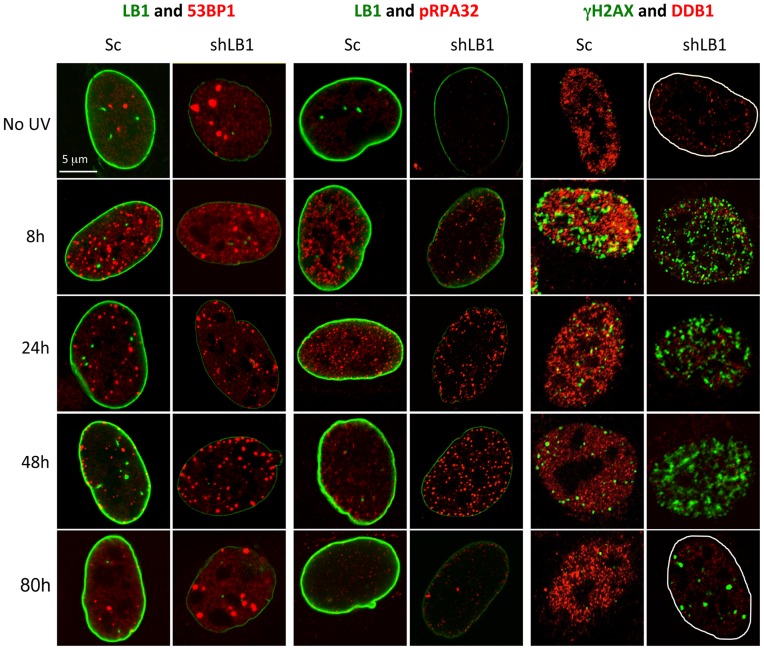
Silencing LB1 expression in U-2 OS cells dramatically delays detection and repair of DNA damage induced by UV. Silenced and control cells were irradiated with 20 J/m^2^ UV, fixed and stained at 8, 24, 48 and 80 hr with antibodies to LB1 (green) and 53BP1 (red); LB1 (green) and pRPA32 (red); and γH2AX (green) and DDB1 (red). No UV samples were from the same transfections. The borders of the nuclei were marked in white in the far right panels. Images of single representative nuclei are shown.

In order to determine whether the delay in DNA repair was due the loss or decrease of NER associated factors, we measured the levels of DDB1, CSB, pRPA32, γH2AX and 53BP1 before and at time intervals after UV irradiation. LB1 silencing induced increased expression and post-translational modification of 53BP1 in non-irradiated cells (ct lanes, Fig. 4), suggesting a DNA stress response to a reduction of LB1. Furthermore, UV irradiation of LB1 silenced cells did not induce an increase in 53BP1 expression like that seen in control cells [35], [37]. Both DDB1 and CSB protein expression levels were decreased in LB1 silenced cells compared to control cells without irradiation (Fig. 4). These results suggest that LB1 silencing alone affected the initiation steps of both NER sub-pathways. The accumulation of phosphorylated pRPA32, which binds to the single stranded region opposite the nucleotide lesion during repair [27], [30], [33] was induced by UV. However silenced cells exhibited both a delay in and lower expression level of pRPA32 compared to control cells (Fig. 4). Interestingly, as expected γH2AX was transiently induced between 0 and 8 hours and was not detectable by 24 hours after UV irradiation in control cells. In contrast, γH2AX was induced between 0 and 8 hours in LB1 silenced cells and persisted until at least 48 hours after UV irradiation (Fig. 4 and 5). Taken together these data show that the levels of DNA damage repair factors involved in NER are significantly decreased in LB1 silenced cells. The lack of sufficient repair factors in LB1 silenced cells could explain the delayed response to the DNA damage caused by UV irradiation.

Because of the delayed NER response in LB1 silenced cells, we analyzed the expression of these and other key factors involved in NER [36] by qRT-PCR of RNA isolated from cells 3 days after LB1 silencing (see Table 1). The activation of p53 suggested by the increase in p53 levels in silenced cells (Fig. 2) was confirmed by the significant increase in mRNA levels for *TP53* (p53) and its effector gene *CDKN1A* (p21) (Table 1). The mRNA levels of two NER factors, *DDB1* and *ERCC6* (CSB), were significantly decreased by more than two-fold compared to control cells. The mRNA levels of other factors involved in NER such as DDB2, ERCC8 (CSA), *XPA, RPA,* and *ERCC5* (XPG) were not significantly altered when comparing LB1 silenced and control cells Table I). In contrast, the expression of *PCNA* and *POLH* (Pol eta), the gene products of which are required for trans-lesion synthesis (TLS) [37]–[39] were significantly down regulated in LB1 silenced cells. The decrease in *DDB1* and *PCNA* mRNA levels in silenced cells is consistent with the decreased protein levels in these cells (Fig. 2 and 4).

**Table 1:** Relative expression analysis of genes associated with NER.

Gene Symbol	Definition	Fold change	*p* value
TP53	Tumor protein p53	1.83	0.017*
CDKN1A	Cyclin-dependent kinase inhibitor 1A	2.3	0.003*
RPA32	Replication Protein A	0.85	0.31
H2AX	H2A histone family, member X	1.21	0.17
PCNA	Proliferating Cell Nuclear Antigen	0.34	0.006*
POLH	Polymerase (DNA directed), eta	0.45	0.004*
DDB1	Damage-specific DNA Binding Protein 1	0.091	0.0001*
DDB2	Damage-specific DNA Binding Protein 2	0.62	0.0527
ERCC8	Excision Repair Cross-Complementing Rodent Repair Deficiency, Complementation Group 8 (CSA)	0.73	0.061
ERCC6	Excision Repair Cross-complementing rodent repair deficiency, Complementation group 6 (CSB)	0.42	0.015*
XPA	Xeroderma Pigmentosum, complementation group A	0.82	0.077
ERCC5	Excision Repair Cross-complementing rodent repair deficiency, Complementation group 5 (XPG)	0.83	0.098

Expression analysis of NER, cell cycle regulation and DNA damage detection factors in LB1 silenced and control cells. mRNA from Sc and shLB1 U-2 OS cells was prepared at 3 days after silencing and analyzed by qRT-PCR using GAPDH as a reference gene. The change in expression of a specific gene was considered significant if the “fold change” was higher than 1.7 or lower than 0.6.

### LB1 silencing causes a delayed initiation of DNA damage repair foci in response to UV irradiation

The mRNA and protein analyses of factors involved in the DNA damage response suggested that some aspects of the NER pathway might be delayed or absent in LB1 silenced cells. Therefore we monitored the timing of the formation of 53BP1, pRPA32 and γH2AX foci, common components of both GG-NER and TC-NER, in the nuclei of control and LB1 silenced cells following exposure to UV. Immunofluorescence analyses confirmed that LB1 silenced cells are deficient in DDB1 before and after UV irradiation (Fig. 5; see Fig. 4). Both 53BP1 and pRPA32 foci formed rapidly in control cells (Sc) within the first 8 hr after UV (Fig. 5 and Figure S3A and B). However, in LB1 silenced cells the number of positive nuclei for both markers was significantly lower compared to controls at this time post-irradiation (Fig. 5; Figure S3A and B). In contrast, more than 63% of both control and silenced cells had γH2AX foci by 8 hrs after irradiation (Figure S3C). However, consistent with the protein analysis (Fig. 4), γH2AX foci persisted in more than 60% of LB1 silenced nuclei until 48 hr after UV, while their presence was significantly reduced in control nuclei as soon as 24 hr after UV (Fig. 5; Figure S3C).

The number of control cells with 53BP1, pRPA32 and γH2AX foci decreased significantly by 48 hr after irradiation (Fig. 5 and Figure S3) as expected for a normal DNA damage repair response [32]–[36], . This is also consistent with removal of CPDs and a high percentage of cell survival (Fig. 3). However, the number of LB1 silenced cells with all three types of foci remained significantly higher than control cells at 48 hr after irradiation. These silenced cells also had a significantly higher incidence of TUNEL positive nuclei, implying the accumulation of double strand breaks that could contribute to apoptosis of these cells (Figure S4 and Fig. 3). By 80 hrs, the majority of surviving LB1 silenced cells retained persistent large γH2AX foci (Fig. 5), suggesting that LB1 silencing affected the resolution of DNA damage foci even after the repair of UV-induced damage.

## Discussion

In this study, we show that decreasing the levels of LB1 in human tumor cell lines by shRNA-mediated silencing leads to a G1 cell cycle arrest. The arrested cells have defects in UV-induced NER that include the delayed formation of repair foci and the removal of the damaged DNA. LB1 silenced cells are highly sensitive to UV irradiation induced apoptosis, most likely due to defects in the cell's ability to mount a timely DNA damage response. We present evidence that the defects in NER are due to the downregulation of some of the protein factors required for the recognition of DNA damage and the formation of repair complexes.

Other evidence for defects in DNA damage repair due to lamin dysfunction has come from studies of Hutchinson Gilford Progeria Syndrome (HGPS) patient cells with the most common LA mutation (G608G) and cells from mice lacking the Zmpste24 protease [42], [43]. Wild type LA is normally processed from a pre-LA precursor by carboxyl terminal farnesylation followed by removal of a terminal peptide containing the lipid moiety [44]. In HGPS, the protease cleavage site is missing due to aberrant splicing, which removes a 50 amino acid segment of the protein containing the Zmpste24 cleavage site [45]. This leads to an excess of permanently farnesylated LA termed progerin that has been related to a constitutively activated DNA damage response, as indicated by an increase in the numbers of 53BP1 foci and increases in phosphorylation of both CHK1 and H2AX [5], [7], [46]. The Zmpste24 null mice.

(*Zmpste24^−/−^*) express elevated levels of pre-LA and are deficient in repairing double strand breaks, in particular homologous repair. This is reflected in their response to ionizing radiation and their increased genomic instability in the absence of radiation. Interestingly, the *Zmpste24^−/−^* MEFs and HGPS fibroblasts also exhibit delayed recruitment of DNA damage response proteins and compromised DNA repair due to defective recruitment of 53BP1 to sites of DNA damage following ionizing radiation [5], [7], [46].

Our finding that LB1 silenced U-2 OS cells are slow to assemble DNA repair complexes is likely attributable to a loss of factors required for NER, which may attenuate the repair of the UV induced DNA lesions. This in turn could lead to the persistent activation of 53BP1, ATR, and p53 triggering a cell cycle arrest at early G1. Alternatively the G1 cell cycle arrest caused by LB1 silencing in non-irradiated cells could cause the persistent activation of ATR in the absence of DNA damage [47]. Further evidence that lamins are involved in regulating ATR comes from the finding that either the expression of LA mutants that cause progeria or the silencing of LA expression by shRNA, causes the ubiquitin mediated degradation of ATR [43]. Nuclear lamina defects due to the accumulation of farnesylated LA have also been shown to trigger an ATM- and NEMO-dependent activation of NF-κB in the absence of DNA damage [48]. Together these findings suggest a possible role for the lamins or lamina structure in regulating DNA damage sensors in cells.

The delayed activation of NER in LB1 silenced cells is associated with the down regulation of factors required for the response to UV. The expression of several genes notably *PCNA*, *POLH* (Pol eta), *DDB1* and *ERCC6* is decreased in silenced cells relative to controls at both mRNA and protein levels. Other factors such as *H2AX*, *RPA*, *ERCC5* (XPG), *ERCC8* and *XPA* are not significantly changed in LB1 silenced cells compared to controls, however the induction and recruitment of these proteins to the damaged sites after UV irradiation was slower in silenced cells. These findings suggest that the delayed response to UV damage caused by LB1 silencing is due to the down-regulation of key factors in both the pre-incision phase of NER, such as DDB1 and CSB, and the post-incision phase, such as PCNA and Pol eta (Fig. 4B and 5) [32]. Thus it appears that both global-and transcription coupled–NER are affected by altering the levels of LB1. In addition, the elevated and extended induction of γH2AX (Fig. 4 and Fig. 5) following irradiation may reflect an increased frequency of double strand breaks due to a delay of NER and failure in trans-lesion synthesis [37], [38], [49]. This increase in double strand breaks may contribute to the increase in apoptosis of LB1 silenced cells following UV damage. It is also important to note that in these cells we detected induction and post-translational modification of 53BP1 and the formation of 53BP1 foci. This finding also suggests that LB1 silenced cells have persistent DNA damage signaling [50], [51] similar to senescent fibroblasts, in which LB1 expression is down regulated [17], [18].

Our findings suggest that LB1 plays an important role in orchestrating transcriptional regulation of various genes involved in DNA damage repair. In human fibroblasts, approximately one-third of the genome is organized into large sharply demarcated regions called lamin associated domains (LADs) that are largely transcriptionally inactive [52]. Since LADs are known to associate with LB1, it is likely that decreasing the LB1 levels by silencing alters these LADs, and therefore gene activity. In support of this, several studies have recently shown that perinuclear positioning of genes and the silencing of chromatin at the nuclear periphery involves complexes of lamins with transcription repressor proteins and histone deacetylases [53], [54]. In addition, the silencing of LB1 expression in tumor cells has been linked to a decrease in RNA Pol II activity [20], [55]. However, additional experiments are required to determine if LB1 is acting by directly regulating the transcription machinery or by defining active and inactive chromosome regions.

## Supporting Information

Figure S1 Transient silencing of LB1 rapidly induces growth arrest in various tumor cell lines. (*A*) Protein level of LB1 was analyzed by immunoblotting at days 3 and 5 after electroporation with vector encoding shRNA targeting LB1 (shLB1). The expression levels of LB1 are relative to the expression of LB1 in control cells transiently expressing shRNA scrambled sequence (Sc) at day 5. (B) Growth rate of shLB1 and Sc cells were compared for 5 days following silencing [n = 3, P≤5.4×10^−6^]; error bars represent standard deviations. (TIF)

Figure S2 Transient silencing of LB1 does not cause DNA damage. An increase in DNA damage as assayed directly by TUNEL was not detected at day 3 following LB1 silencing. Similarly, no differences in levels of γH2AX or pRPA32 were detected between control (Sc) and LB1 silenced cells (shLB1). Error bars represent standard deviations [n = 4; number of examined nuclei >500; TUNEL p = 0.12; γH2AX p = 0.089; pRPA32 p = 0.071]. (TIF)

Figure S3 Quantitation of the 53BP1 (A), pRPA32 (B) and γH2AX (C) positive Sc and shLB1 cells at 8, 24, 48 and 80 hours following 20 J/m^2^ UV irradiation are presented in lower panel. Error bars represent standard deviations [n = 3; number of analyzed nuclei in each experiment was >600; example of positive nuclei presented in Figure 5]. (TIF)

Figure S4 FACS analysis DNA damage assayed by TUNEL in control (Sc) and LB1 silenced cells (shLB1) at 24, 48 and 80 hr following UV irradiation (20 J/m^2^). The data was calibrated to positive control cells treated with DNase I as described. (TIF)
