# Supplementary figures and images for: Regulation of Nucleotide Excision Repair by Nuclear Lamin B1
Source: PLoS One. 2013 Jul 24;8(7):e69169. doi: 10.1371/journal.pone.0069169 (PMC3722182; doi:10.1371/journal.pone.0069169)

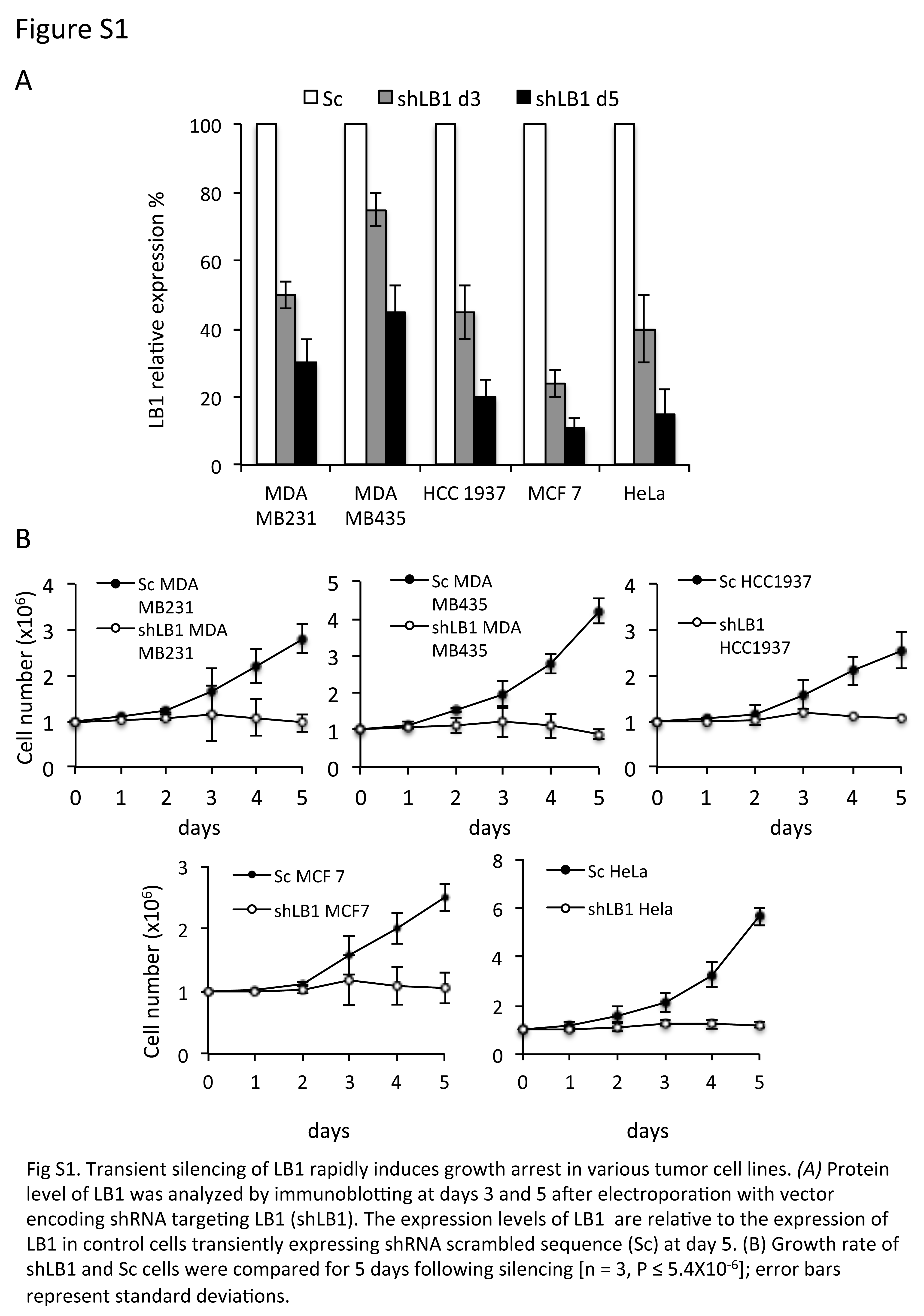

Supplement: Figure S1 — (TIF) [file pone.0069169.s001.tif]

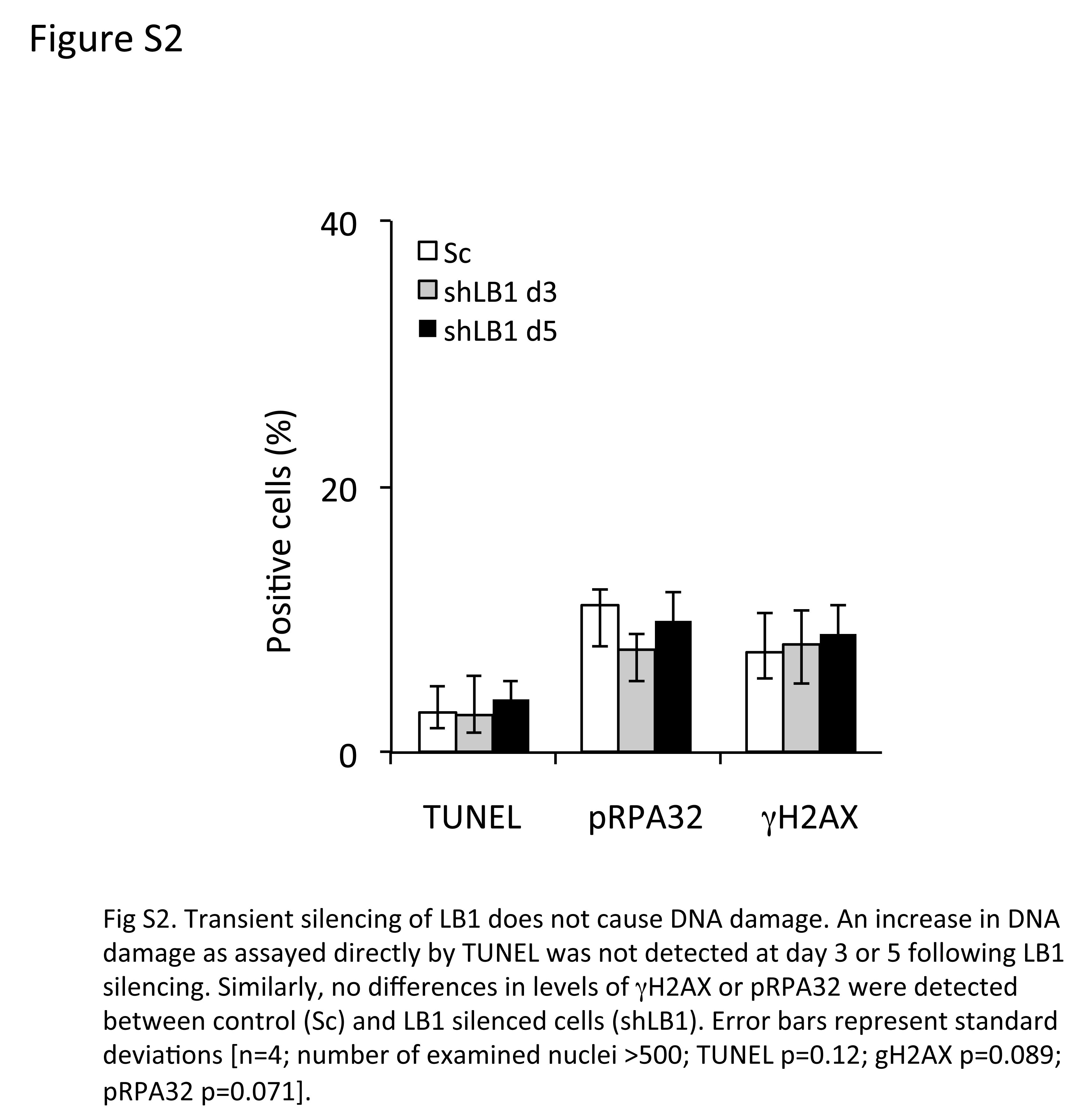

Supplement: Figure S2 — (TIF) [file pone.0069169.s002.tif]

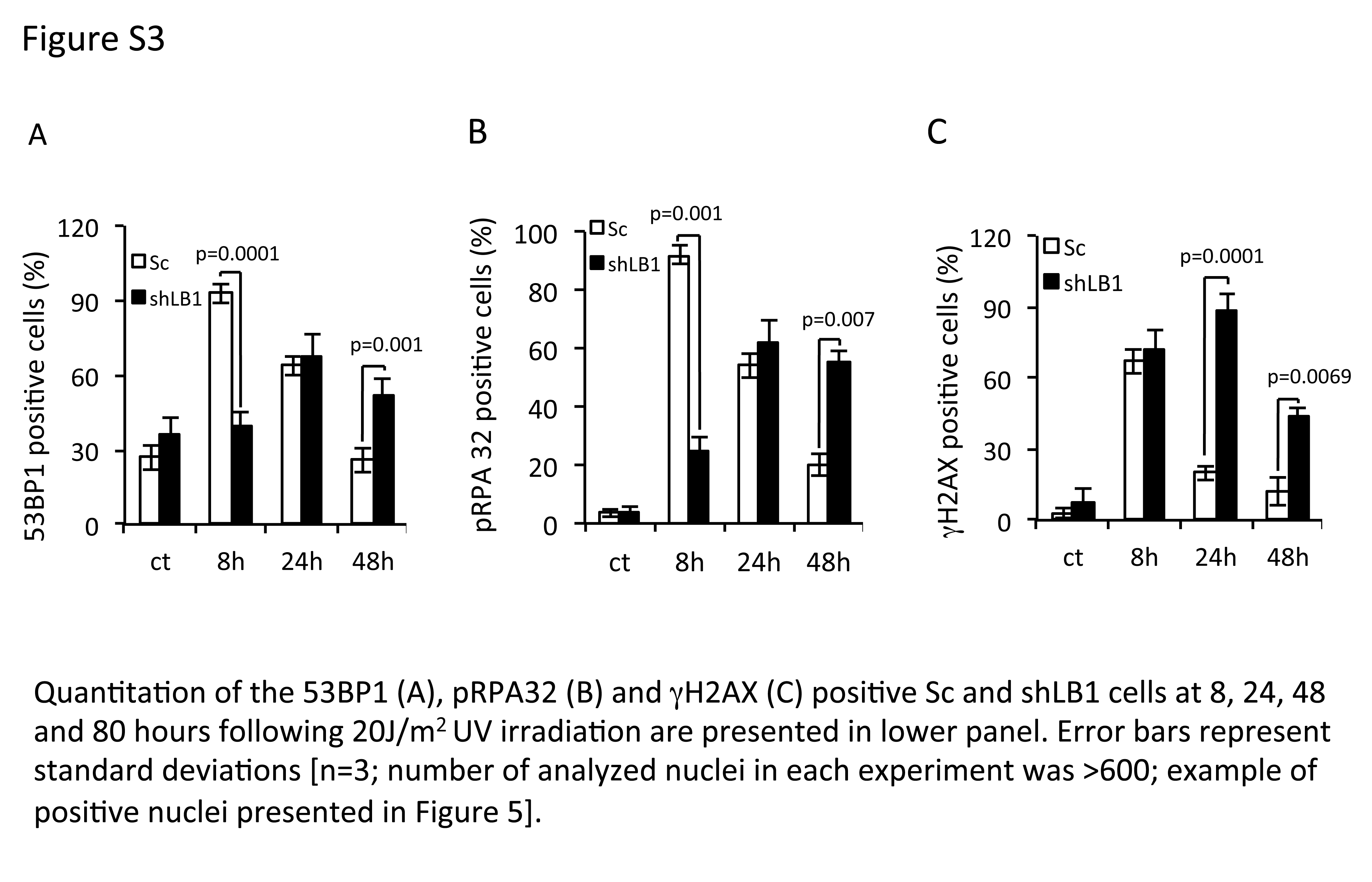

Supplement: Figure S3 — (TIF) [file pone.0069169.s003.tif]

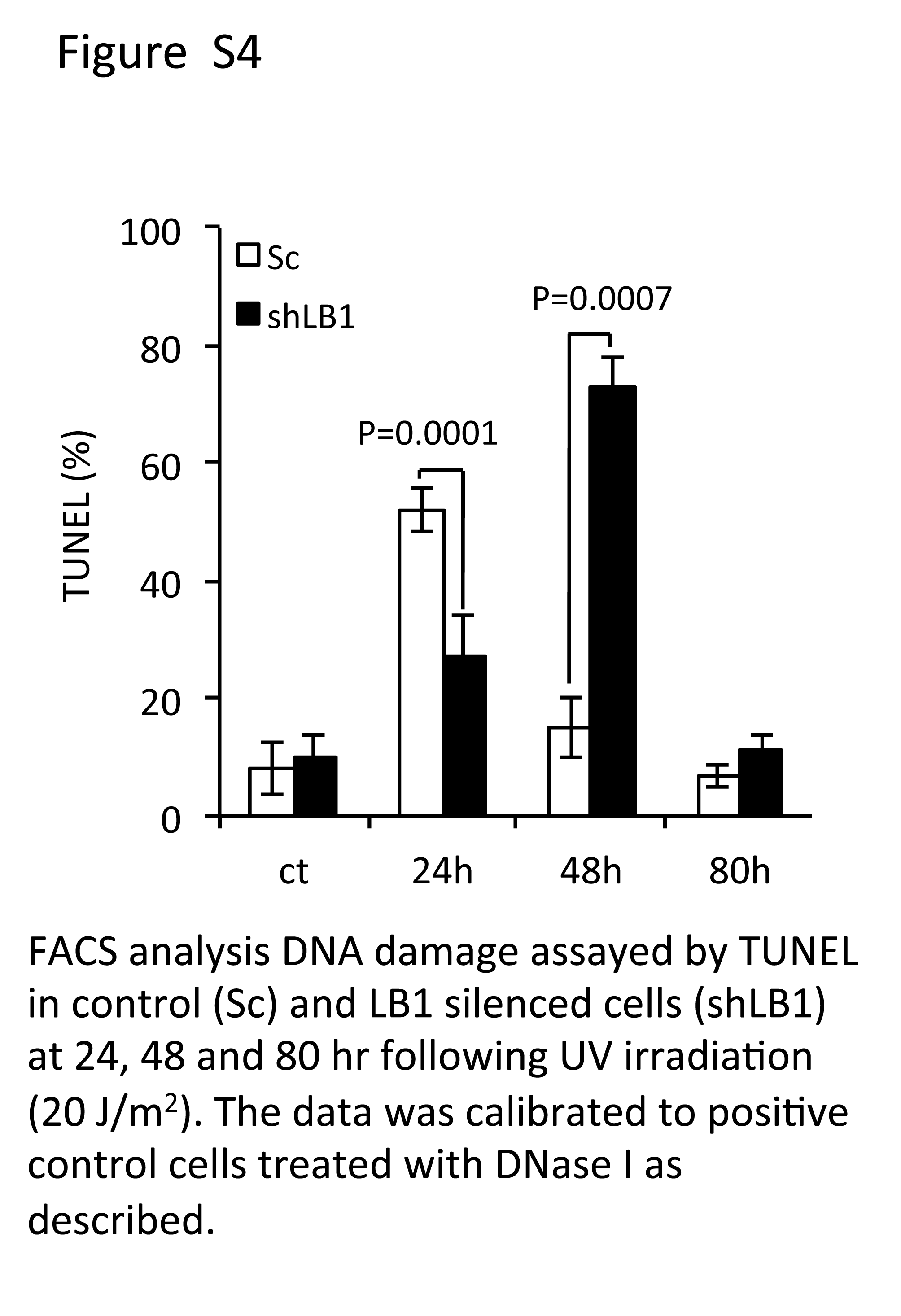

Supplement: Figure S4 — (TIF) [file pone.0069169.s004.tif]
